# A study on appetite of overweight/obese patients with type 2 diabetes mellitus based on multimodal magnetic resonance imaging

**DOI:** 10.3389/fnimg.2025.1615654

**Published:** 2025-07-15

**Authors:** Mingxuan Gao, Liya Gong, Yanmei Zeng, Dongling Li, Junyan Wen, Ying Guo, Zhujia Li, Jingwen Luo, Chunling Chen, Ge Wen

**Affiliations:** Nanfang Hospital, Southern Medical University, Guangzhou, China

**Keywords:** obesity, type 2 diabetes, magnetic resonance imaging, neuroimaging, fMRI

## Abstract

**Purpose:**

To investigate the alterations of brain structure and function in brain regions related to ingestive desire in overweight/obese T2DM patients, and the correlation with clinical data.

**Subjects:**

52 patients with overweight/obese type 2 diabetes mellitus (T2DM group), 62 patients with simple obesity (OB group), and 40 healthy subjects (HC group).

**Assessment:**

By means of gray matter morphometric indices (cortical thickness, surface area, etc.), resting-state functional magnetic resonance indices (ALFF, ReHo, FC) and DTI eigenvalues (AD, MD, etc.).

**Statistical tests:**

Comparisons among the three groups were made using one-way ANOVA, bonferroni *post hoc* test for two-way comparisons, and spearman for correlation analysis.

**Results:**

Compared with the OB and HC groups, the T2DM group showed a significant reduction in cortical thickness in the bilateral superior frontal gyrus, inferior frontal gyrus orbital region, and the lower part of the right middle frontal gyrus, and the functional connectivity of the prefrontal cortex showed a significant trend of enhancement. Meanwhile, compared with the HC group, the T2DM group showed a significant decrease in FA (fractional anisotropy) values in the midline region of the orbitofrontal cortex bilaterally, and the left inferior frontal gyrus orbital region also showed a significant decrease in FA values, whereas AD (axial diffusivity), MD (mean diffusivity), and RD (radial diffusivity) increased significantly.

**Data conclusion:**

T2DM patients have significant alterations in gray matter structure, brain white matter integrity and brain function, and most of the brain regions with significant differences are in the prefrontal cortex, which confirms that the abnormal desire to ingest in T2DM patients is closely related to the functional alterations of the reward system, and that observing the brain function and structural changes of the reward loop through imaging may help in the early diagnosis and treatment of overweight/obese T2DM patients.

## Introduction

The global prevalence of obesity and type 2 diabetes mellitus (T2DM) has reached alarming levels, and epidemiological studies continue to emphasize their synergistic metabolic and cardiovascular risks. Notably, more than 90% of patients with type 2 diabetes are associated with overweight or obesity, emphasizing the critical role of dysregulated energy intake and obesity in disease progression (Reed et al., 2021). A key driver of this imbalance is aberrant food craving-a complex neurobehavioral phenomenon influenced by sensory, cognitive, and reward-related neural circuits. Evidence suggests that increased food cue reactivity (FCR), particularly in response to olfactory and visual stimuli, may exacerbate caloric overconsumption and weight gain, thereby perpetuating metabolic dysfunction in T2DM populations (Perszyk et al., 2023).

Neuroimaging studies have begun to reveal the neural basis of food cravings, including the role of areas such as the pyriform cortex, insula, and orbitofrontal cortex in sensory representation and reward processing. For example, enhanced olfactory imagery is associated with stronger FCR and longitudinal increases in body mass index (BMI), which are mediated by heightened activation of olfactory and limbic networks (Perszyk et al., 2023). Similarly, dysregulation of the basolateral amygdala in response to food cues has been associated with susceptibility to weight gain (Burke and Small, 2016). However, existing studies have largely relied on single-modality neuroimaging approaches (e.g., functional MRI), which may overlook the interplay between structural, functional, and white matter alterations in the brain of obese T2DM patients.

Emerging multimodal MRI techniques that integrate functional connectivity, gray matter volume measurements, and brain white matter fiber analysis provide a comprehensive framework for dissecting these interactions. In addition, cognitive-behavioral analyses have revealed different eating patterns and misperceptions in obese individuals, which may be related to neural features that inhibit the control of impaired or overactive reward pathways. Despite these advances, no study has yet applied multimodal MRI to investigate the neurobehavioral correlates of food cravings in overweight/obese T2DM populations, a gap that limits targeted interventions.

The present study aimed to address this critical knowledge gap by exploring the neural mechanisms of food craving in overweight/obese type 2 diabetic patients using multimodal MRI. We hypothesized that aberrant connectivity in the sensory reward network and structural alterations in prefrontal regulatory areas would be associated with elevated FCR and disordered eating behaviors. This study uses multimodal neuroimaging technology, combined with clinical data and behavioral evaluation, to analyze the neural circuit characteristics of feeding desire and its interaction with metabolic abnormalities in overweight/obese T2DM patients. The specific objectives include: (1) comparing the differences in brain activation and brain structure between overweight/obese T2DM patients, simple obesity patients, and healthy controls; (2) Analyze the correlation between brain structure, brain function, white matter, and glucose and lipid metabolism indicators related to feeding behavior; (3) Explore the heterogeneity of central feeding regulatory networks in patients with different levels of blood glucose control. The research results are expected to provide new evidence for revealing the neurobiological basis of food loss control in overweight/obese T2DM patients, and lay a theoretical basis for developing metabolic intervention strategies based on brain targets, such as neurofeedback training and deep brain stimulation.

## Materials and methods

### Subjects

In accordance with the Declaration of Helsinki, this study was approved by the Ethics Committee of local hospital. Patients with overweight/obese type 2 diabetes mellitus and patients with simple obesity attending the outpatient clinic of local hospital between September 2023 and September 2024 were recruited into this study, as well as healthy subjects matched for age, gender, and education level. All participants participated in this study after having sufficient judgment, understanding informed consent and signing a written informed consent form, and all studies were conducted in accordance with relevant guidelines and regulations.

Inclusion criteria for the T2DM group: (1) age 18–65 years; (2) type 2 diabetes mellitus diagnosed by endocrinologists according to WHO diabetes mellitus diagnostic and classification criteria (1999); (3) receiving conventional glucose-lowering drug therapy during the disease; (4) right-handed; (5) signing an informed consent. Exclusion criteria: (1) with contraindications to MRI scanning; (2) patients with secondary obesity; long-term use of medications affecting body weight; (3) comorbidities with severe organic diseases such as heart/brain, liver, kidney, etc.; (4) comorbidities with heavy mental disorders such as schizophrenia. (5) Taking antidepressants or any drug that affects the functional activity of the brain.

Enrollment criteria for the OB group: (1) age 18–65 years; (2) BMI ≥ 24 kg/m^2^; (3) subjects were right-handed; (4) signed informed consent; Exclusion criteria: (1) with contraindications to MRI scanning; (2) patients with secondary obesity; long-term use of medications affecting body weight; (3) comorbidities of serious organic diseases such as heart, brain, liver, kidney, and so on; (4) comorbidities of heavy mental disorders such as schizophrenia. (5) Taking antidepressants or any drug that affects the functional activity of the brain.

Inclusion criteria of HC group: (1) age 18–65 years; (2) BMI 18.5–23.9 kg/m^2^; (3) Not meeting the WHO diagnostic criteria for diabetes mellitus; (4) Normal results of somatic and laboratory examinations; (5) No history of taking any glucose-lowering related medications, no history of any psychiatric disorders, and no history of drug or alcohol abuse; (6) Signing the informed consent form. Exclusion criteria: (1) with contraindications to MRI scanning; (2) patients with secondary obesity such as hypothyroidism, Cushing’s syndrome, etc.; long-term use of medications affecting body weight; (3) combined with serious organic diseases such as heart, brain, liver, kidney, and so on; (4) people with family history of psychiatric illnesses and other family histories related to induced psychiatric illnesses.

Diagnosis was determined by cross-consultation between two or more senior endocrinologists (associate physicians and above). Basic information was collected from all participants, including name, age, gender, educational background, current health status, family history, disease duration, and key data such as waist circumference, hip circumference, and body mass index (BMI). A self-administered general data and clinical characteristics collection form was used. On the day of the MRI, all participants were asked to arrive at the hospital at 8:00 am after an overnight fast. Blood samples were collected by venipuncture to determine clinical parameters including fasting blood glucose (FPG), glycosylated hemoglobin (HbA1c), and cholesterol levels (i.e., triglycerides, total cholesterol, LDL, cholesterol, and HDL, cholesterol).

### Appetite related scale

#### 100 mm appetite visual analog scale (VAS)

This questionnaire was designed using the Visual Analog Scale (VAS), which is a widely recognized international instrument with high reliability and validity. Its main scope of application covers the assessment of five dimensions: appetite, hunger perception, satiety, satisfaction and thirst of the subjects. Underneath each question item is a 10-centimeter long line, with the two extremes of the line representing the most positive and the most negative ratings. Subjects were asked to mark a vertical line on the line to reflect their actual feelings in the current moment. The researcher used a ruler to measure the distance from the left end of the line to the point marked by the participant as a basis for evaluation.

#### Control of eating questionnaire (COEQ)

The COEQ consists of 21 items divided into six sections, which subjects are required to complete based on how they have felt over the past seven days. The first two sections (Q1, Q2) contain questions about the general level of appetite and overall mood (independent of food cravings); the third and fourth sections (Q3-Q12) contain questions assessing the frequency and intensity of food cravings in general; the fifth section (Q13-Q18) deals with cravings for specific diets (e.g., dairy, starchy, sweet, or non-sweet); and finally the sixth section (Q20, Q21) assesses the individual’s level of perceived control over the refusal of specified, craved foods. A 100-mm visual analog rating scale (VAS) was used to assess 20 items.

The above questionnaires were completed by the same clinical physician before each participant underwent MRI examination.

### Magnetic resonance data acquisition

The MRI scans of all subjects in this study were done in the imaging department of local hospital. MRI examinations were performed on all subjects, prior to which we calibrated the magnetic resonance equipment for data stability. During the scanning process, the subjects were placed supine on the examination bed and the head position was stabilized by using head restraint straps and foam pads to minimize the interference of head movements on the MRI signal acquisition. Prior to the start of the examination, it was explained to the subject that he/she should keep his/her eyes closed and relaxed throughout the examination, avoid falling asleep, maintain consciousness, and minimize unnecessary mental activity. The scanning equipment was a GE 3.0 T MRI scanner (SIGNA Architect, GE Medical System, the United States) with a 48-channel standard head coil with the following parameters: Using the brain volume scanning sequence, we acquired structural information on 3D-T1-weighted images (3D-T1WI), a process achieved by a 3D magnetization-prepared rapid gradient echo sequence (MP-RAGE) with the following parameters: Flip Angle (FA) = 15°, Inversion Time (TI) = 1,000 ms, Slice Thickness = 1 mm, Space Between Slice = 1 mm, Repetition Time (TR) = 2,338.3 ms, Echo Time (TE) = 3.112 ms, Number of Slices = 392, FOV = 256 mm × 256 mm, matrix = 512 × 512, bandwidth = 122 Hz/Px, voxel size 0.5 mm × 0.5 mm × 0.5 mm. The BOLD imaging technique utilizes the Spin Echo-Echo Plane Imaging (SE-EPI) method of a spin-echo sequence. TR/TE = 2000/30 ms, FA = 90°, layer thickness = 3 mm, layer spacing = 4 mm, 1 mm interval, FOV = 220 × 220 mm, matrix = 64 × 64, the whole brain area was covered by 32 layers of axial images, and 180 volume data were acquired continuously by axial scanning. DTI sequence: TR/TE = 10,000/70 ms, FA = 90°, FOV = 256 × 256 mm, matrix = 256 × 256, 1 acquisition signal, layer thickness = 2 mm, number of layers = 70, no interval acquisition, combined with parallel acquisition technique to reduce image deformation, and applied diffusion-sensitive gradient (b = 1000s/mm^2^) in 64 non-collinear directions for data acquisition.

### Image analyses

#### Structural image

For the preprocessing of 3D-T1WI structural data, we used the Freesurfer 7.2.0 image analysis software platform, aiming to achieve cortical and subcortical nuclei precise segmentation of the cortex and subcortical nuclei and extraction of relevant quantitative parameters. The preprocessing process started with the conversion of all raw high-resolution 3D-T1WI data. Freesurfer constructs a vertex-based model of the cortical surface, which defines cortical thickness based on the distance from the gray and white matter boundaries to the soft meninges. The automated cortical processing process performed by the “recon-all” script covers the following stages: motion correction and average brain image generation, removal of non-cerebral tissue using a hybrid watershed algorithm and surface deformation, implementation of an automated Talairach transformation, segmentation of the subcortical white matter and gray matter volume structures, implementation of intensity normalization and automated topology correction, and implementation of the intensity normalization and topology correction. We perform intensity normalization and automatic topology correction, expansion and smoothing, spherical mapping and alignment, reconstruction of the cortical surface, cortical segmentation and morphological evaluation. This series of steps was performed according to Freesurfer’s standardized mean cortical surface template (i.e., FSaverage template) and nonlinear procedures. Once the data were processed, we implemented strict quality control of the results for each subject. For the subjects’ data whose automated processing did not meet the standard, we made manual adjustments and applied the script template again to the calculations to ensure the accuracy and reliability of the data. Data based on Desikan-Killiany-Tourville (DKT) were extracted from the files created by the previous “recon-all” script in Freesurfer via the script “aparcstats2table,” including thickness (mm), surface area (mm^2^), gray matter volume (mm^3^) and curvature (mm^−1^).

#### Functional images

The pre-processing step of BOLD-fMRI data was realized with the help of SPM12 toolbox and the DPARSF component of DPABI 8.2 toolbox. This process covers from data format conversion (i.e., converting DICOM format data to NIFTI format), including format conversion, removal of the first 10 unstable time points, time layer correction, head movement correction, cranial stripping, spatial normalization based on the DARTEL algorithm, linear drift removal, and regression covariates (including the Friston24 head movement parameter, white matter signal, and cerebrospinal fluid signal). In addition, subjects with excessive head movements were excluded (subjects with head movements greater than 3 mm or 3° in any frame), and filtering was taken to limit the frequency range of the analysis at a later stage of the preprocessing process, followed by smoothing (Gaussian kernel = 4 × 4 × 4 mm^3^) to reduce noise and improve spatial continuity of the data.

Using age and gender as covariates, the data from the preprocessed BOLD-fMRI were imported into DPARSF of the DPABI software toolbox, and the resting-state functional MRI metrics ALFF and ReHo were calculated for each subject.

According to previous literature, in this study, bilateral thalamus, anterior ventral nucleus of thalamus, posterior posterior lateral ventral, ventral tegmental area, superior frontal gyrus, middle frontal gyrus, inferior frontal gyrus, orbital frontal cortex, insula, cingulate, striatum, nucleus ambiguus, amygdala, substantia nigra, blueprints, and clumps in which the alterations in resting-state functional indices were detected during the study were selected as the seed points, and the ROIs of the region of interest (ROIs) were produced by the Marsbar toolbox, and then by the DPABI toolbox of the DPARSF software to calculate FC maps between ROIs and whole-brain voxels. The correlation coefficient r was obtained by calculating the average time series of each region of interest (ROI) and correlating it with the time series of other voxels in the whole brain of the subjects, and in order to improve the normality analysis of the data, the correlation coefficients were transformed from Fisher’s to z to obtain the z-score matrix, and the z-score values were used in the next step of statistical analysis.

#### Diffusion tensor imaging analysis

Following the standard procedure, diffusion data and white matter fiber bundle imaging were processed using DSI studio 2024 software as follows:

(1) Data in DICOM file format were imported into the DSI studio software and converted to SRC file format, and to ensure the accuracy of data processing, the B-meter was checked using an automatic quality control program; (2) pre-processing steps such as de-head movement, eddy current correction, and removal of non-native brain tissues were performed on individual diffusion images using the FSL toolbox, and diffusion data were reconstructed using Q-space difference reconstruction (QSDR), with the diffusion sample length ratio set to 1.25; (3) whole brain white matter fiber bundles were outlined using an automated tracking strategy based on map-guided trajectory identification to improve its reproducibility, with diffusion maps of each subject normalized to MNI space by nonlinear alignment, and the generated trajectories were compared with HCP-1065 fiber tracking atlases at Hausdorff distances to determine which ones most closely matched the target structures. Deterministic fiber tracking was generated using a random seed strategy with the following parameter settings: the Hausdorff distance tolerance (tolerance to individual bundle variation) = 16 mm was set by default, the variation threshold was 20%, the angular threshold was randomly selected from 15–90°, and the step size was randomly selected from 0.5 voxels to 1.5 voxels, with tracks of lengths less than 15 mm or greater than 150 mm being discarded, and a total of 50,000 seeds were placed.

Regions with significant BOLD-fMRI differences were selected as regions of interest to explore their structural connectivity. All ROIs were expanded by a voxel into the white matter so that they were in contact with the fibers, and metrics such as mean fractional anisotropy (FA), mean diffusion coefficient value (MD), and fiber length were extracted for all the subject fibers by deterministic fiber tracking of fiber bundles from each pair of symmetric ROIs.

### Statistical analyses

The statistical software SPSS 27.0 was utilized for the analysis, with Levene’s variance chi-square test being performed on all indexes prior to analysis. When the variance did not conform to a chi-square distribution, Welch’s test was employed. Gender comparisons among the three groups were conducted using the chi-square test, with *p* < 0.05 denoting a statistically significant difference. Clinical data, gray matter morphology indexes, rs-fMRI indexes, and DTI eigenvalues were analyzed using one-way ANOVA. When the ANOVA results differed, two-by-two comparisons were made using the Bonferroni post-hoc test. A statistically significant difference was indicated by *p* < 0.05. To ensure the robustness of the findings, fMRI analyses were performed with age and gender as covariates. To correct for single voxel *p* < 0.001 and cluster size *p* < 0.05 differences, a Gaussian random field (GRF) multiple comparisons correction was implemented. Following this correction, statistically significant brain regions were identified using a two-tailed correction. Spearman’s correlation analysis was then employed to examine the relationship between gray matter morphology data, fMRI correlations, and DTI eigenvalues, along with the clinical information. Finally, use SPSS Power Analysis to analyze the actual observed effect size (such as *η*^2^ or Cohen’s * f * for ANOVA, R^2^ change in regression analysis), actual sample size, and set significance level (*α* = 0.05), and calculate the *post hoc* statistical test power of the main findings of this study.

## Results

### Analysis of clinical data

Initially, 67 patients with overweight/obese type 2 diabetes mellitus (T2DM), 70 patients with simple obesity (OB), and 47 healthy subjects were recruited, and 30 patients with large head movements, artifacts in images, and incomplete data were excluded. The study finally included 52 patients with overweight/obese type 2 diabetes mellitus (T2DM group), 62 patients with simple obesity (OB group), and 40 healthy subjects (HC group). The clinical statistics of the three groups are shown in Table 1.

**Table 1:** Statistical table of clinical data of the three groups.

Measures		T2DM (*n* = 52)	OB (*n* = 62)	HC (*n* = 42)	F	*P* value
Sex	Male	39 (75.0%)	23 (37.1%)	17 (42.5%)	17.937	<0.001*#
	Female	13 (25.0%)	39 (62.9%)	23 (57.5%)		
Age		37.06 (8.12)	32.29 (7.16)	29.80 (7.13)	10.712	<0.001*#
BMI (kg/m^2^)		30.90 (4.80)	30.92 (5.83)	22.54 (2.92)	43.825	<0.001#^
WHR		0.97 (0.06)	0.91 (0.08)	0.83 (0.09)	43.730	<0.001^*#^^
FPG (mmol/L)		8.19 (2.96)	5.23 (0.68)	5.14 (0.39)	48.702	<0.001^*#^
HbA1c (%)		9.27 (2.74)	6.15 (1.05)	5.49 (0.17)	67.600	<0.001^*#^^
TP (g/L)		72.32 (5.59)	76.00 (4.89)	74.51 (3.74)	8.081	0.002^*^
ALB (g/L)		43.82 (4.39)	45.31 (3.62)	47.20 (3.00)	10.018	<0.001^#^^
ALT (U/L)		58.80 (53.27)	44.12 (34.96)	21.96 (12.37)	10.311	<0.001^#^^
AST (U/L)		39.14 (38.58)	26.59 (14.62)	18.82 (5.12)	8.266	<0.001^#^^
TC (mmol/L)		5.44 (1.10)	5.16 (0.92)	4.87 (0.84)	3.936	0.022^#^
TG (mmol/L)		3.48 (4.84)	2.35 (3.11)	1.36 (1.04)	4.271	0.002^#^
LDL-C (mmol/L)		3.58 (0.83)	3.40 (0.83)	3.10 (0.79)	3.742	0.026^#^
HDL-C (mmol/L)		1.01 (0.26)	1.15 (0.26)	1.46 (0.34)	29.589	<0.001^*#^^
UA (μmol/L)		403.32 (109.32)	397.96 (97.68)	388.63 (97.82)	0.237	0.789
UREA (mmol/L)		4.33 (1.19)	4.35 (0.96)	5.13 (1.04)	8.133	<0.001^#^^
CREA (μmol/L)		67.68 (18.73)	71.58 (14.70)	72.08 (15.43)	1.092	0.338
Appetite		3.75 (2.91)	5.98 (2.30)	3.75 (2.84)	9.82	<0.001*^
Hunger		3.13 (2.85)	4.54 (2.45)	3.04 (2.89)	4.15	0.018*^
Fruit		3.96 (2.78)	5.52 (2.53)	5.78 (2.50)	7.09	0.001*#
Starchy foods		3.90 (2.63)	5.23 (2.43)	5.19 (2.52)	4.74	0.010*#

The data is displayed as the mean (standard deviation), “*” indicates the comparison between T2DM group and OB group (*p* < 0.05); “#” indicates the comparison between T2DM group and HC group (*p* < 0.05); “^” indicates the OB group is *p* < 0.05 compared with the HC group.

**Figure 1:**
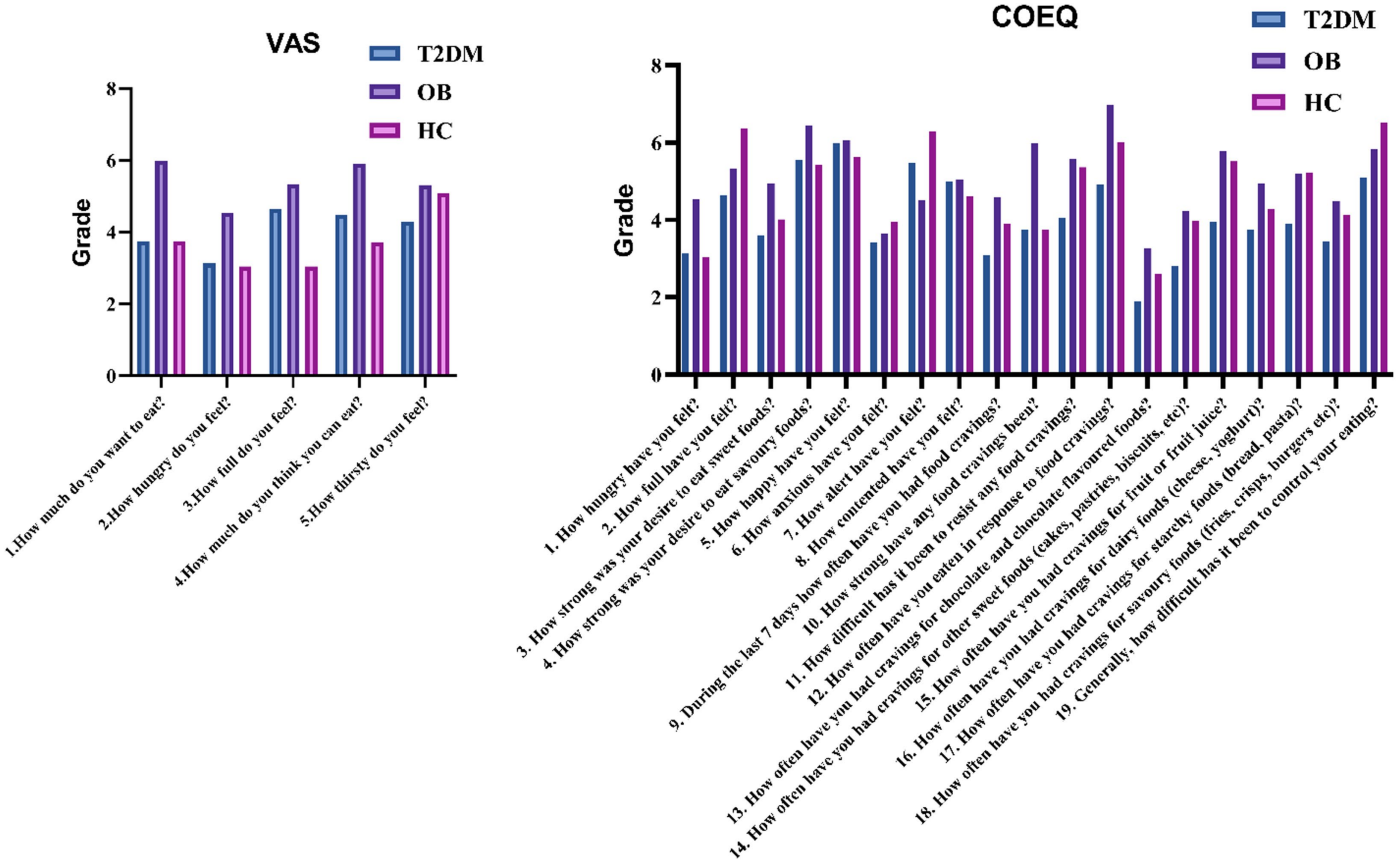
Comparison of VAS and COEQ scales among the three groups.

Comparative analysis of the demographic data of the three groups revealed that the proportion of males in the T2DM group was significantly higher than that in the OB and HC groups, and the age was significantly higher than that in the OB and HC groups; the BMI was not significant in the T2DM group and the OB group, but it was higher than that in the HC group in both groups; and the waist-to-hip ratio in the T2DM group was significantly higher than that in the OB and HC groups. In terms of biochemical index statistics, FPG and HbA1c showed a decreasing trend in the T2DM, OB and HC groups; in addition, compared with the HC group, ALT, AST, TC, TG and LDL-C were significantly higher in the T2DM group, and ALB, HDL-C and UREA were significantly lower in the T2DM group; compared with the HC group, HbA1c, ALT and AST in the OB group were higher than that of the HC group, and the above were statistically different (*p* < 0.05). The statistics of appetite-related scale scores of the three groups are shown in Figure 1. The appetite, hunger, fruit preference and starchy food preference scores of the T2DM group were lower than those of the OB group, and the fruit craving scores and starchy food cravings were lower than those of the HC group; and the scores of appetite and hunger of the OB group were higher than those of the HC group. Finally, all of the above were statistically different (*p* < 0.05).

### Structural magnetic resonance imaging

**Figure 2:**
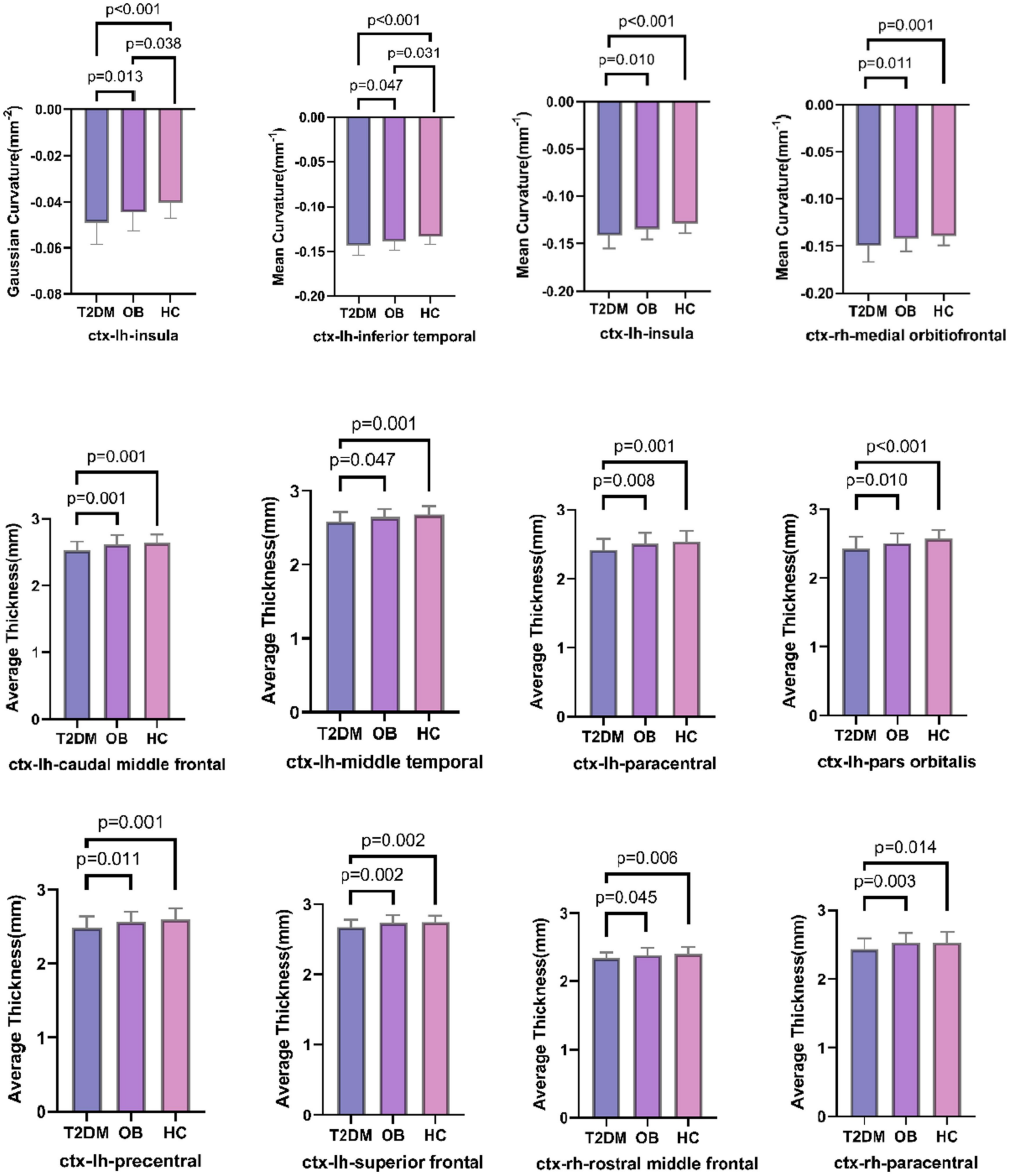
Comparison of three groups of brain regions with differences in gray matter morphological indices; lh: left hemisphere, rh: right hemisphere.

The results of the analysis of the gray matter morphological data of the structural images are shown in Figure 2. The brain regions with significant differences among all three groups were the left insula and the left inferior temporal gyrus; the absolute mean Gaussian curvature of the cortex of the left insula and the absolute mean curvature of the left inferior temporal gyrus in the T2DM group were significantly more compared with those in the OB group and the HC group; and the absolute mean curvature of the cortex in the midline portion of the cortex of the left insula and right orbitofrontal cortex in the T2DM group was significantly more. However, the cortical thickness of the bilateral superior frontal gyrus and paracentral lobule, the caudal part of the left middle cingulate gyrus, the precentral gyrus, the middle temporal gyrus, the orbital part of the inferior frontal gyrus, and the lower part of the right middle frontal gyrus in the T2DM group was significantly reduced, and all of the above results were corrected by Bonferroni (*p* < 0.05).

### Functional magnetic resonance imaging

**Figure 3:**
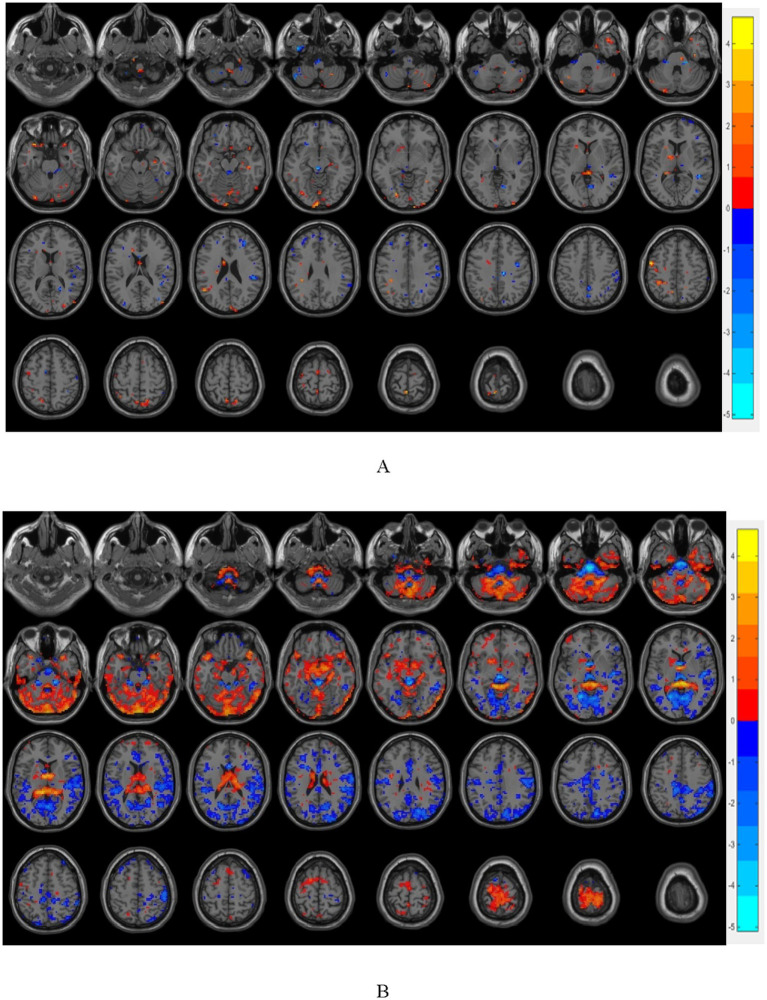
**(A)** shows the brain regions with differences in FC between T2DM group and OB group, and **(B)** shows the brain regions with differences in FC between T2DM group and HC group (*p* < 0.05, GRF corrected); warm color indicates that the strength of functional connection is increased, and cold color indicates that the strength of functional connection is reduced.

The brain regions with significant differences in resting-state magnetic resonance indexes compared among the three groups are shown in Table 2 and Figure 3. Compared with the OB and HC groups, ALFF was significantly increased in localized clusters in the medial left superior frontal gyrus, left middle frontal gyrus, right superior frontal gyrus, and right precentral gyrus cortex in the T2DM group; and ReHo was significantly increased in localized clusters in the right superior frontal gyrus, left middle temporal gyrus, left amygdala, and right orbital infra-orbital frontal gyrus cortex in the T2DM group, and the results of the above were statistically significant differences (*p* < 0.05, corrected by GRF).

**Table 2:** Brain regions with differences in ALFF and ReHo in three groups.

Measure	AAL	BA	MNI (X Y Z)	Cluster size (mm^3^)	Peak T value
ALFF	Frontal_Sup_Medial_L	BA10_L	−15 63 3	378	4.0672
	Frontal_Mid_L	BA10_L	−30 60 6	243	4.2287
	Precentral_R	BA6_R	57 9 36	324	4.1391
	Frontal_Sup_R	BA8_R	18 30 60	135	3.775
ReHo	Frontal_Sup_R	BA6_R	21 12 66	135	4.4181
	Temporal_Mid_L	BA21_L	−51 -21 -3	243	5.6400
	Amygdala_L	BA30_L	−30 0–24	108	4.1551
	Frontal_Inf_Orb_R	BA11_R	18 15–24	135	3.9788

**Figure 4:**
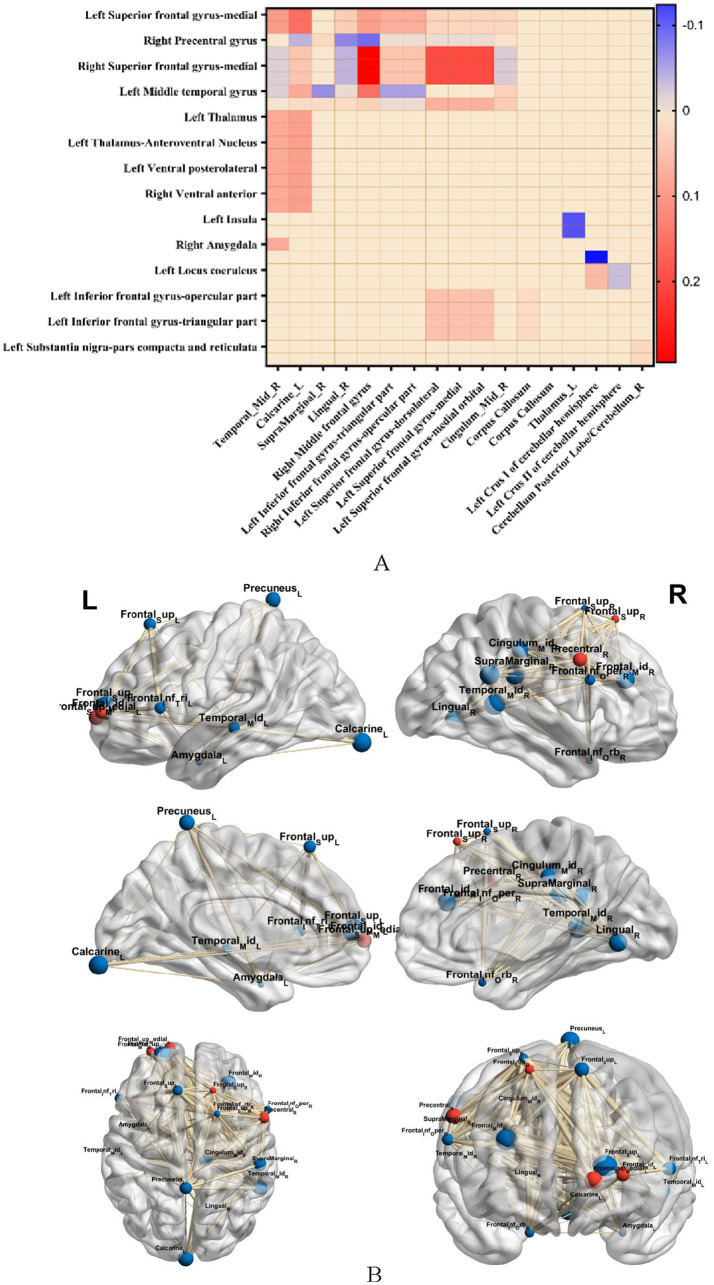
**(A)** Represents the comparison of brain functional connectivity strengths with significant differences in FC among the three groups (*p* < 0.05, GRF corrected), warm color indicates FC connectivity strength, and cold color indicates reduced FC strength; **(B)** represents BrainNet software visualization of brain regions with significant differences in FC among the three groups, and red color indicates clumped ROIs with altered resting state metrics.

Brain regions related to ingestive reward (thalamus, anterior ventral nucleus of thalamus, posterior ventral lateral, ventral tegmental area, superior frontal gyrus, middle frontal gyrus, inferior frontal gyrus, orbital frontal cortex, insula, cingulate, striatum, nucleus accumbens, amygdala, substantia nigra, and blue-spot) and clumps of altered functional indicators of the resting state were selected as the seed points (ROIs) and the functional brain connectivity that had a significant difference of FC among the three groups are presented in Figure 4.

Compared with the OB and HC groups, in the T2DM group, (1) the medial left superior frontal gyrus with the right middle temporal gyrus, left talar fissure, and right limbic supramarginal gyrus, the right superior frontal gyrus with the right middle frontal gyrus and left talar fissure, the bilateral anterior ventral nuclei and posterior lateral thalamic nuclei with the left subfrontal gyrus deltoid and the left inferior frontal gyrus capping, the bilateral anterior ventral and left superior frontal gyrus, the dorsal-lateral superior frontal gyrus and the corpus callosum bilaterally, and the bilateral inferior subfrontal gyrus deltoid and inferior frontal gyrus capping with the corpus callosum, and left superior frontal gyrus; (2) significant enhancement of FC in the right precentral gyrus with the right lingual gyrus, right middle frontal gyrus, right supramarginal gyrus, left middle temporal gyrus with the right middle cingulate gyrus, left amygdala with the right middle temporal gyrus, bilateral insulae with the left thalamus, bilateral amygdala with the left brainstem/medulla oblongata, dorsal-lateral frontal gyrus with the dorsal part of the dorsolateral frontal gyrus and the cerebellar hemispheres of the left calvarium I, bilateral bluish pallidum with the cerebellar hemispheres of the left calvarium I and the cerebellar hemispheres of the left calvarium II, bilateral nigral substantia nigra dense and reticular formation with the posterior cerebellar lobe/right cerebellum had significantly lower FC, all of which were statistically different from each other (*p* < 0.05, GRF corrected).

### Diffusion tensor imaging

Based on the results that there were significant differences in resting-state functional indexes among the three groups in comparison, we selected bilateral superior frontal gyrus, lateral orbitofrontal cortex, midline orbitofrontal cortex, and orbitofrontal gyrus as the ROIs of interest for the DTI analysis. Deterministic fiber tracing was performed in DSI studio software, and all ROIs were expanded by one voxel to the white matter to make contact with the fiber bundles, and based on the QSDR reconstruction algorithm High-definition nerve fiber images of all ROIs were obtained, and the FA, MD, AD, and RD values of each ROI were obtained, as shown in Table 3.

**Table 3:** Comparison of DTI metrics across three groups of areas of interest.

Measures	Brain region	T2DM	OB	HC	P
FA	ctx-lh-medial orbitofrontal	0.34 ± 0.04	0.35 ± 0.03	0.37 ± 0.03	0.013^#^
	ctx-rh-medial orbitofrontal	0.38 ± 0.04	0.38 ± 0.05	0.41 ± 0.05	0.002^#^^
	ctx-lh-lateral orbitofrontal	0.35 ± 0.03	0.35 ± 0.03	0.37 ± 0.03	<0.001^#^^
	ctx-lh-pars orbitalis	0.35 ± 0.03	0.35 ± 0.03	0.36 ± 0.32	0.031^#^
MD	ctx-lh-lateral orbitofrontal	0.86 ± 0.05	0.85 ± 0.04	0.83 ± 0.04	0.003^#^^
(10^−3^ mm^2^/s)	ctx-lh-pars orbitalis	0.83 ± 0.05	0.82 ± 0.04	0.79 ± 0.04	<0.001^#^^
AD (10^−3^ mm^2^/s)	ctx-lh-pars orbitalis	1.13 ± 0.04	1.12 ± 0.04	1.10 ± 0.04	<0.001^#^^
RD	ctx-lh-lateral orbitofrontal	0.71 ± 0.05	0.69 ± 0.05	0.66 ± 0.05	<0.001^#^^
(10^−3^ mm^2^/s)	ctx-lh-pars orbitalis	0.68 ± 0.05	0.67 ± 0.05	0.64 ± 0.05	<0.001^#^^
	ctx-lh-medial orbitofrontal	0.79 ± 0.09	0.77 ± 0.07	0.74 ± 0.06	0.014^#^
	ctx-rh-medial orbitofrontal	0.71 ± 0.07	0.70 ± 0.08	0.67 ± 0.06	0.014^#^

The data is displayed as the mean (standard deviation), “*” indicates the comparison between T2DM group and OB group (*p* < 0.05); “#” indicates the comparison between T2DM group and HC group (*p* < 0.05); “^” indicates the OB group is *p* < 0.05 compared with the HC group.

Compared with the HC group, the MD and RD values of the lateral left orbitofrontal cortex, and the AD, MD, and RD values of the left inferior frontal gyrus orbital region were increased in the T2DM group; the FA values of the lateral left orbitofrontal cortex, the left inferior frontal gyrus orbital region, and the bilateral orbitofrontal cortical midline regions, and the RD values of the bilateral orbitofrontal cortical midline regions were decreased; and the MD and RD values of the lateral left orbitofrontal cortex and the left inferior frontal gyrus orbital region, and the RD values of the left superior frontal gyrus orbital fiber bundles were increased in the OB group compared to the HC group; there were statistically significant differences between these values. AD, MD, and RD values of the orbital fiber bundles in the left inferior frontal gyrus were increased; FA values of the lateral left orbitofrontal cortex were decreased; all of the above were statistically different (*p* < 0.05).

### Correlations

**Figure 5:**
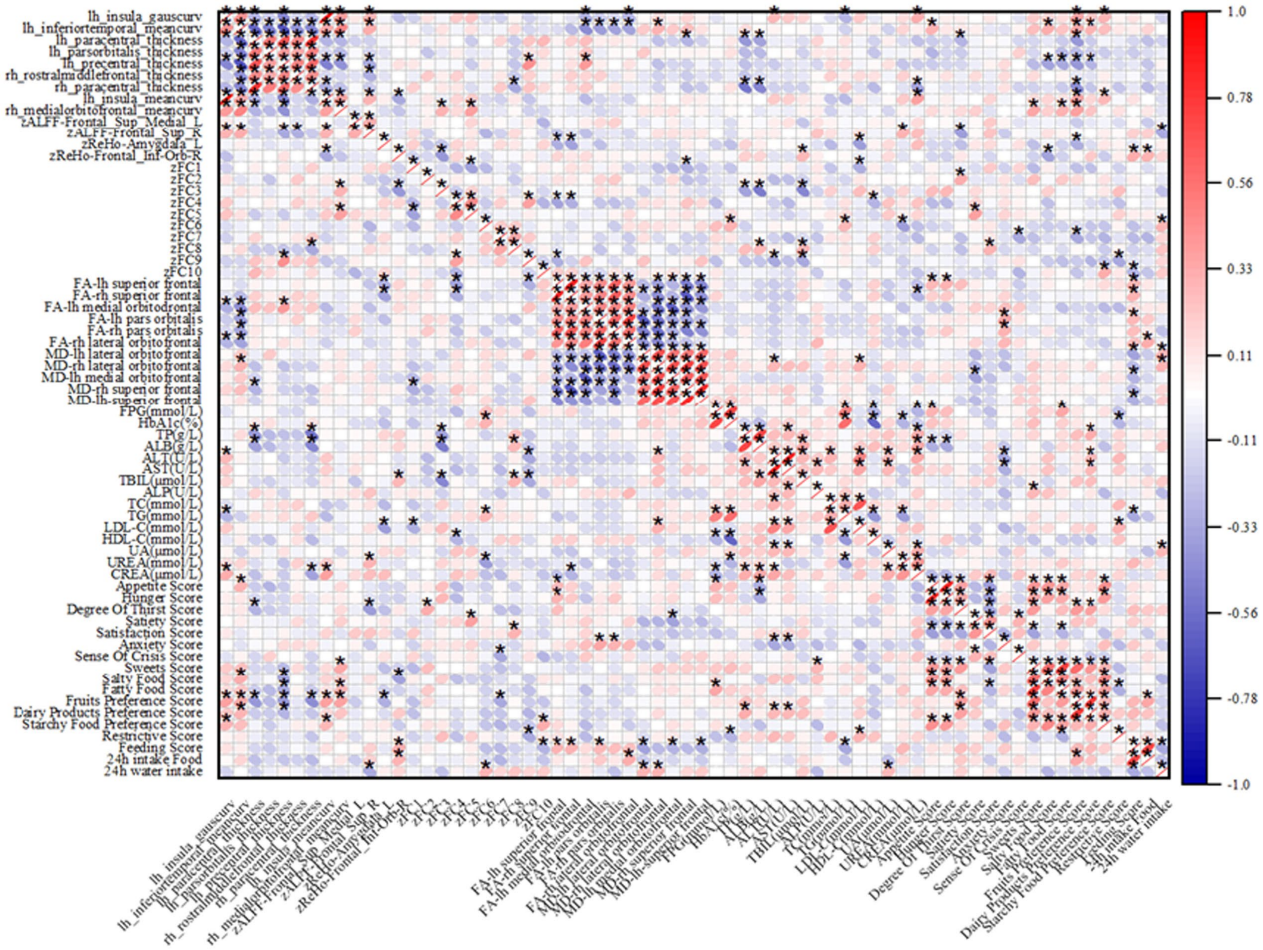
Heatmap of correlation between imaging indices and clinical data, red indicates positive correlation, blue indicates negative correlation; “*” indicates *p* < 0.05.

In the T2DM group, there were significant correlations between several imaging indices and clinical data, as shown in Figure 5. For biochemical indices, the thickness of the cortex of the lower right middle frontal gyrus and the right paracentral lobule was positively correlated with LDL-C; ALFF enhancement of localized clumps of the cortex in the medial part of the left supraoptic gyrus was negatively correlated with LDL-C; and the AD value of the lateral cortex of the right orbital frontal, the AD value of the orbit of the left infraorbital gyrus, were positively correlated with HbA1c were positively correlated.

Regarding the appetite scale, in the T2DM group, ReHo enhancement of cortical curvature at the midline of the left insula and right orbitofrontal cortex, and localized mass in the left amygdala cortex were negatively correlated with fruit preference scores; ALFF enhancement of localized mass in the medial cortex of the left supraorbital gyrus was negatively correlated with appetite scores; ReHo enhancement of localized mass in the cortex of the right infraorbital gyrus was positively correlated with appetite scores; ReHo enhancement of localized ReHo enhancement of clumps was negatively correlated with salty food preference scores; FA values in the left superior frontal gyrus were positively correlated with appetite scores and thirst scores; and MD values in the midline section of the left orbitofrontal cortex were negatively correlated with satiety scores.

## Discussion

Recent studies have shown that overweight/obese T2DM patients have significant cortical structural remodeling, brain functional alterations, and brain white matter damage, especially in brain regions related to ingestive desire and reward processing, and that these changes may serve as an important biological basis for abnormal ingestive behaviors by affecting neural signal integration and loop function. In this study, we elucidated the brain regions with abnormal ingestive desire in overweight/obese T2DM patients by integrating high-resolution 3D-T1WI, rs-fMRI and DTI. The results of the study explored the multimodal MRI characterization and mechanism of ingestive desire from three dimensions: brain structural changes, brain functional changes and brain white matter microstructural changes.

Several studies have confirmed that prefrontal cortex (PFC) thickness is significantly reduced in overweight/obese T2DM patients, especially in the dorsolateral prefrontal (dlPFC) and orbitofrontal cortex (OFC) (García-Casares et al., 2014). In the present study, we found by high-resolution 3D-T1WI that the brain regions with reduced cortical thickness in overweight/obese T2DM patients compared to non-T2DM controls were mainly concentrated in the prefrontal lobe, with increased absolute cortical curvature observed in the left insula, left inferior temporal gyrus, and right orbitofrontal cortex at the midline, in line with the results of previous studies. Previous studies revealed a correlation between elevated insulin levels and reduced frontal cortical thickness, indicating a negative correlation between frontal cortical thickness and insulin levels (Sasaki et al., 2001). The frontal lobe is a highly important region within the default mode network, with a pivotal role in the resting brain and extensive structural and functional connections with other brain regions (Okabayashi et al., 2015).

In this study, the brain regions with reduced cortical thickness in the T2DM group included bilateral superior frontal gyrus, paracentral lobule, caudal left middle cingulate gyrus, precentral gyrus, middle temporal gyrus, and orbital portion of the inferior frontal gyrus, most of which were located in the region of the reward loop, and the curvature of the cortex in the midline portion of the right orbitofrontal cortex was negatively correlated with the fruit preference scores, which is in line with the study of the previous scholars. Toniolo et al. found that the prefrontal cortex and the frontal orbital gyrus cortical thickness and volume were negatively correlated with drug addiction intake and food intake that contribute to obesity (Toniolo et al., 2018). Structural abnormalities in the orbitofrontal cortex, a key brain region for integrating reward value and decision making, may lead to overvaluation of caloric foods. In the present study, the thickness of the orbitofrontal cortex (OFC) was reduced in overweight/obese T2DM patients compared with that in obesity-only patients and healthy controls, and the reduced thickness of the cortex was negatively correlated with fruit preference scores in the appetite scale, suggesting that the structural damage may diminish the ability to inhibit the urge to ingest food. This result supports the “reward prediction error hypothesis” proposed by Schultz et al. that reduced OFC thickness may reduce the ability to accurately encode the reward value of food, resulting in the need for patients to overeat to compensate for the attenuation of dopamine signaling (Schultz et al., 1997). In addition, the reduced strength of the structural covariation network of the OFC with the nucleus accumbens (NAc) may further disrupt reward prediction error signaling and induce impulsive feeding (Drucker, 2022). This structural alteration may stem from chronic hyperglycemia-induced oxidative stress, resulting in reduced neuronal dendritic spine density (Moran et al., 2019).

Notably, the mean cortical curvature of the left insula was significantly increased in absolute value in the T2DM group compared with the OB and HC groups, and the cortical curvature of the left insula was negatively correlated with the fruit preference score, which is in line with the previous findings of Craig et al. suggesting that alterations in insula cortical curvature may impair satiety perception by interfering with endoreceptor signaling integration (Craig, 2009); Eunice Y Chen et al. found that reduced insula cortex thickness in overweight/obese T2DM patients was significantly associated with visceral fat area and fasting leptin levels (Chen et al., 2020). Structural damage to the insula, which plays a key role in taste and appetite regulation as an endosensory signal processing center, may impair the precision of perception of hunger/satiety signals, and patients with reduced insula thickness have a weakened neural response to gastric hunger hormone at rest, which may lead to increased unconscious eating behavior (Schulz et al., 2023).

It was found by rs-fMRI that ALFF was significantly increased in localized clusters of the medial left superior frontal gyrus, left middle frontal gyrus, right superior frontal gyrus and right precentral gyrus cortex in the T2DM group, and ReHo was significantly increased in localized clusters of the right superior frontal gyrus, left middle temporal gyrus, left amygdala, and right orbital infra-orbital frontal gyrus cortex in the T2DM group, in contrast to the OB group and the HC group, which play a significant role in the modulation of appetite, reward mechanisms, and emotion regulation These areas play an important role in the regulation of appetite, reward mechanisms and emotion regulation.

Elevated ALFF values usually indicate enhanced spontaneous activity in local brain regions, which may be associated with cognitive control or emotion regulation, and abnormally elevated ALFF values in the prefrontal lobe may be associated with impaired inhibitory control during ingestive behaviors. The ReHo values reflect the synchronization of neuronal activity in local brain regions, and their abnormalities may affect cognitive integration and behavioral control. Similar reports have been reported in previous studies, Cui et al. (2014) observed that the left middle frontal gyrus ReHo values of T2DM patients showed a significant trend of elevated values using the analysis of ALFF versus ReHo, suggesting that the brain structure and function can be reorganized and compensatory mechanisms to complete the already impaired neural functions (Hämäläinen et al., 2007). Abnormal ReHo values in prefrontal regions within the Default Mode Network (DMN) are associated with cognitive impairment and may weaken inhibition of food impulses.

In terms of rs-fMRI studies, the present study found that in comparison with the OB and HC groups, the ALFF values of the localized mass in the left middle frontal gyrus cortex were increased in the T2DM group, the FC values of the right superior frontal gyrus with the right middle frontal gyrus, the dorsal lateral frontal gyrus with the corpus callosum bilaterally were elevated, the FC values of the right precentral gyrus with the right middle frontal gyrus were reduced, and the values of the functional connectivity strength of the right precentral gyrus with the right middle frontal gyrus were negatively correlated with the appetite scores, suggesting that that the dorsolateral prefrontal cortex is abnormally active in the overweight/obese T2DM population, which may enable a significant increase in craving for high-calorie foods by inhibiting cortical activity, which is consistent with previous findings that obese individuals have diminished dlPFC activity, resulting in a reduced inhibitory control over food cues (Asplund et al., 2010; Hass et al., 2016). Therefore, the dorsolateral prefrontal cortex has been recognized as a potential therapeutic target that may help improve dietary control in obese patients by enhancing its function (Gluck et al., 2017).

In addition, in the present study, FC values of bilateral thalamus and anterior ventral nucleus and posterior lateral and left inferior frontal gyrus delta were elevated and functional connectivity values were positively correlated with LDL-C, FC values of left amygdala and right middle temporal gyrus, bilateral amygdala and left medulla oblongata, and left middle temporal gyrus and right middle cingulate gyrus were reduced, and functional connectivity between prefrontal cortex and thalamus was significantly enhanced in the resting state, suggesting that hypothalamus-prefrontal loops and metabolic signaling. The hypothalamus-prefrontal loop and metabolic signaling were suggested to be abnormal. The arcuate nucleus of the hypothalamus (ARC) drives foraging behaviors by transmitting hunger signals to the vmPFC via AgRP neurons, which are activated in response to starvation to promote animals’ exploration of food sources, whereas the vmPFC regulates the prioritization of such behaviors by integrating metabolic signals (e.g., leptin and starvation hormone), and according to a previous study, regulating the release of dopamine from the nucleus ambiguus through glutamatergic projections that affects the calculation of food reward value and goal-directed behavior, and blocking glutamatergic transmission in the medial ventral prefrontal cortex disinhibits dopamine release in the nucleus ambiguus, leading to enhanced ingestive behavior (Dolan, 2002). In contrast, chronic high-calorie diets remodel this loop, leading to reduced sensitivity of the vmPFC to metabolic signals and exacerbating overeating (Alcantara et al., 2022; Lowe et al., 2019). The amygdala, a core structure of the limbic system, is involved in ingestion regulation by encoding the emotional potency of food (e.g., pleasure or disgust).

In terms of resting-state brain functional networks, the regions with abnormal rs-fMRI metrics are mainly the default mode network of the brain, i.e., there are still certain regions of the brain that are active in the resting state, and the default network usually has high metabolic activity, which makes it more susceptible to the effects of hyperglycemia. Mechanistically, changes in brain function may be related to insulin resistance and chronic inflammation; insulin not only regulates blood glucose, but also plays a role in the brain in regulating appetite and energy balance; insulin resistance may lead to disruption of insulin signaling pathways in the brain, which in turn affects the regulation of the desire to eat (Fordahl and Jones, 2017; Lyra et al., 2019).

The results of DTI indexes in the present study showed that the white matter integrity of the frontal and orbitofrontal cortex brain regions was significantly impaired in the T2DM group compared with the OB and HC groups, as evidenced by a decrease in the FA value and an increase in the AD, MD, and RD values, and a correlation between the abnormal DTI indexes and the Appetite Scale scores, a finding that is consistent with previous studies of T2DM encephalopathy, suggesting that chronic hyperglycemia This finding is consistent with previous studies of T2DM encephalopathy (Li et al., 2020; Reijmer et al., 2013), suggesting that chronic hyperglycemia and insulin resistance may lead to axonal demyelination and glioblast proliferation through mechanisms such as oxidative stress, inflammatory responses, and disruption of the blood brain barrier. Notably, obesity-related metabolic abnormalities (e.g., lipid deposition and leptin resistance) may further exacerbate white matter injury. In this study, compared with the healthy population, there were significant abnormalities in DTI indexes in the orbitofrontal cortex brain region of patients with simple obesity, which could be supported by combining with the previous studies, and the FA value of the hook fascicle was negatively correlated with BMI, suggesting that the visceral fat accumulation may be caused by the release of pro-inflammatory factors (e.g., TNF-*α*, IL-6) to directly damage white matter fibers (Verstynen et al., 2013; García-García et al., 2022; Emanuela et al., 2012). In addition, the significant elevation of RD values in the orbitofrontal cortex in the T2DM group compared with the HC group suggests that myelin damage may be the main pathological mechanism, whereas the relative stabilization of AD values suggests relative preservation of axonal structures, which is similar to the pathological features of diabetic peripheral neuropathy, suggesting a commonality of damage between the central and peripheral nervous systems (Alotaibi et al., 2022; Qin et al., 2019). In addition, the volume of specific brain regions detected by sMRI, such as the dorsolateral prefrontal cortex, may also serve as a biomarker for predicting the effectiveness of feeding behavior interventions (Godet et al., 2022). Based on sMRI for structural anomaly localization, precise intervention of the prefrontal striatal loop can be achieved through neural regulation techniques such as transcranial magnetic stimulation to improve inhibitory control. Training patients to regulate prefrontal cortex activity through real-time functional MRI neurofeedback can reduce cravings for high calorie foods and improve weight. Studies have shown that neurofeedback targeting the insula can reduce food addiction scores, and the effect can last for 6 months (Karch et al., 2015). MRI based neurofeedback training can improve patients’ dietary behavior by regulating the activity of the prefrontal cortex (PFC). Clinical trials have shown that after 12 weeks of intervention, patients’ craving scores for high sugar and sweet foods decrease (Rebello and Greenway, 2016).

In the present study, we found significant abnormalities in the regulatory network of ingestive desire in overweight/obese T2DM patients by multimodal MRI technique, which were characterized by (1) hyperfunctioning of the reward system; (2) impaired prefrontal regulation; and (3) structural alterations of the hypothalamic-limbic system. It is worth noting that brain function abnormalities are more significantly associated with metabolic disorders in patients with T2DM than in patients with obesity alone, suggesting that hyperglycemic states may exacerbate brain area damage through oxidative stress and neuroinflammation (Indulekha et al., 2011).

## Conclusion

Through the multimodal MRI technical analysis of overweight/obese T2DM patients, it was found that compared with the OB and HC groups, T2DM patients had significant alterations in gray matter structure, cerebral white matter integrity, and brain function. The results of sMRI and rs-fMRI showed that most of the brain areas with gray matter structural alterations and cerebral functional activity and functional connectivity abnormalities in the T2DM group were in the prefrontal cortex, which, in combination with the DTI’s index and correlation fiber tracing, it further confirmed that the abnormal desire to ingest in T2DM patients was closely related to the functional changes of the reward system, and the observation of the brain functional and structural changes of the reward loop in T2DM patients through imaging may help in the early diagnosis and treatment of overweight/obese T2DM patients.

## Data Availability

The datasets presented in this article are not readily available because personal privacy. Requests to access the datasets should be directed to 1207803073@qq.com.
